# Automated scoring for a Tablet-based Rey Figure copy task differentiates constructional, organisational, and motor abilities

**DOI:** 10.1038/s41598-021-94247-9

**Published:** 2021-07-21

**Authors:** Marco A. Petilli, Roberta Daini, Francesca Lea Saibene, Marco Rabuffetti

**Affiliations:** 1 Department of Psychology, University of Milano-Bicocca, Piazza dell’Ateneo Nuovo, 1, 20126 Milan, Italy; 2 NeuroMI-Milan Center for Neuroscience, Milan, Italy; 3 IRCCS Fondazione Don Carlo Gnocchi ONLUS, Milan, Italy

**Keywords:** Diagnostic markers, Brain injuries, Dementia, Movement disorders, Parkinson's disease, Neurological disorders, Stroke, Parkinson's disease, Human behaviour, Cognitive ageing, Motor control, Perception, Visual system

## Abstract

Accuracy in copying a figure is one of the most sensitive measures of visuo-constructional ability. However, drawing tasks also involve other cognitive and motor abilities, which may influence the final graphic produced. Nevertheless, these aspects are not taken into account in conventional scoring methodologies. In this study, we have implemented a novel Tablet-based assessment, acquiring data and information for the entire execution of the Rey Complex Figure copy task (T-RCF). This system extracts 12 indices capturing various dimensions of drawing abilities. We have also analysed the structure of relationships between these indices and provided insights into the constructs that they capture. 102 healthy adults completed the T-RCF. A subgroup of 35 participants also completed a paper-and-pencil drawing battery from which constructional, procedural, and motor measures were obtained. Principal component analysis of the T-RCF indices was performed, identifying spatial, procedural and kinematic components as distinct dimensions of drawing execution. Accordingly, a composite score for each dimension was determined. Correlational analyses provided indications of their validity by showing that spatial, procedural, and kinematic scores were associated with constructional, organisational and motor measures of drawing, respectively. Importantly, final copy accuracy was found to be associated with all of these aspects of drawing. In conclusion, copying complex figures entails an interplay of multiple functions. T-RCF provides a unique opportunity to analyse the entire drawing process and to extract scores for three critical dimensions of drawing execution.

## Introduction

**Figure 1:**
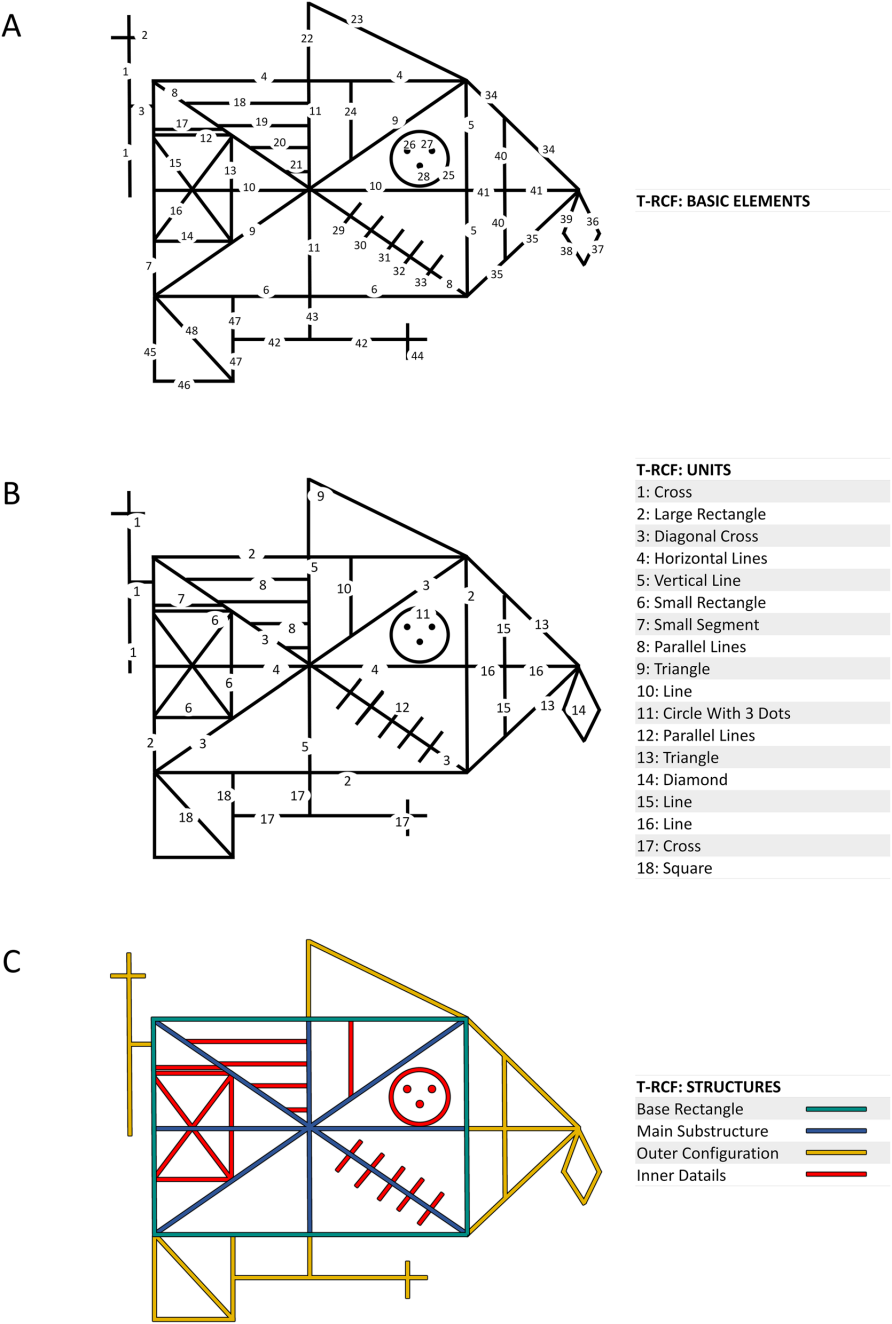
Three classifications of constitutive parts of the T-RCF. (**A**) Division into basic elements: the T-RCF (i.e., Tablet-based Rey complex figure) is divided into 48 basic elements. (**B**) Division into units: the T-RCF is divided into 18 geometrical units according to the classical classification^5^. (**C**) Division into structures: the T-RCF is divided into the 4 main constitutive structures according to the classification of Trojano et al.^63^. The figure is a reproduction of the RCF^5^ created assembling geometric elements with Microsoft PowerPoint (Microsoft Office 365 Version 2105; www.office.com).

"Paper-and-pencil" drawing tasks are primarily adopted for the assessment of visuo-constructional skills, originally defined as the ability to combine one-dimensional units to form two-dimensional models^1^. Typically, drawing tasks require examinees to copy simple geometrical figures or complex designs^2^. However, more complex stimuli are known to put a more significant load on visuospatial functions and consequently have a higher sensitivity in measuring constructional ability, even in healthy participants or patients with mild constructional impairments^3^. The Rey–Osterreith Complex Figure copy task (RCF)^4,5^ (see Fig. 1) is ranked as one of the top 10 tests used by neuropsychologists, and it is the test most commonly employed in the assessment of constructional skills^2,6–8^. It can be used for both research and clinical purposes and can be administered to individuals aged from 6 to 93 years. In this task, examinees are asked to reproduce a complex figure by copying it freehand^4,5^. The level of accuracy in the RCF-copy is calculated as a measure of visuo-constructional ability^2,9^. The Rey–Osterrieth 36-point system^5,10–12^ is the scoring method most widely used to evaluate it (according to the survey of International Neuropsychological Society members by Knight et al.^9^). This system evaluates, on a two-point scale, the degree of copy accuracy for each of the 18 geometric units that constitute the figure (Fig. 1B). This measure has several merits. It has adequate internal consistency^13,14^, test–retest reliability^15,16^ as well as being strongly related to performance in other visuospatial perceptual tests such as line orientation^13^, Visual Reproduction of the Wechsler Memory Scale^13,17^, and Raven's Standard Progressive Matrices^18^. Moreover, in the neuropsychological field, RCF-copy accuracy is recognised as being highly sensitive to the progression of Alzheimer's dementia^3^, and it is useful in detecting damage involving various areas of the brain, including the middle occipital, posterior-parietal, superior temporal, and frontal cortex, more extensively in the right hemisphere^19^.

However, such a high sensitivity comes at a price. Given its complexity, RCF-copy involves numerous and different processes, primarily recruiting constructional, organizational, and motor abilities^2,20^. Consequently, accuracy in the final graphic product is the result of an interplay between various functions. The heterogeneity in cognitive and motor requirement reduces its specificity in measuring pure constructional processes. Moreover, the established scoring methodologies^5,10–12^ are not able to capture the richness of clinical information contained in such a complex task and separate their distinct contribution from the final graphic product (see, for example^21–23^, for studies on the effects of motor or planning impairments on drawing in Parkinson’s disease).

**Figure 2:**
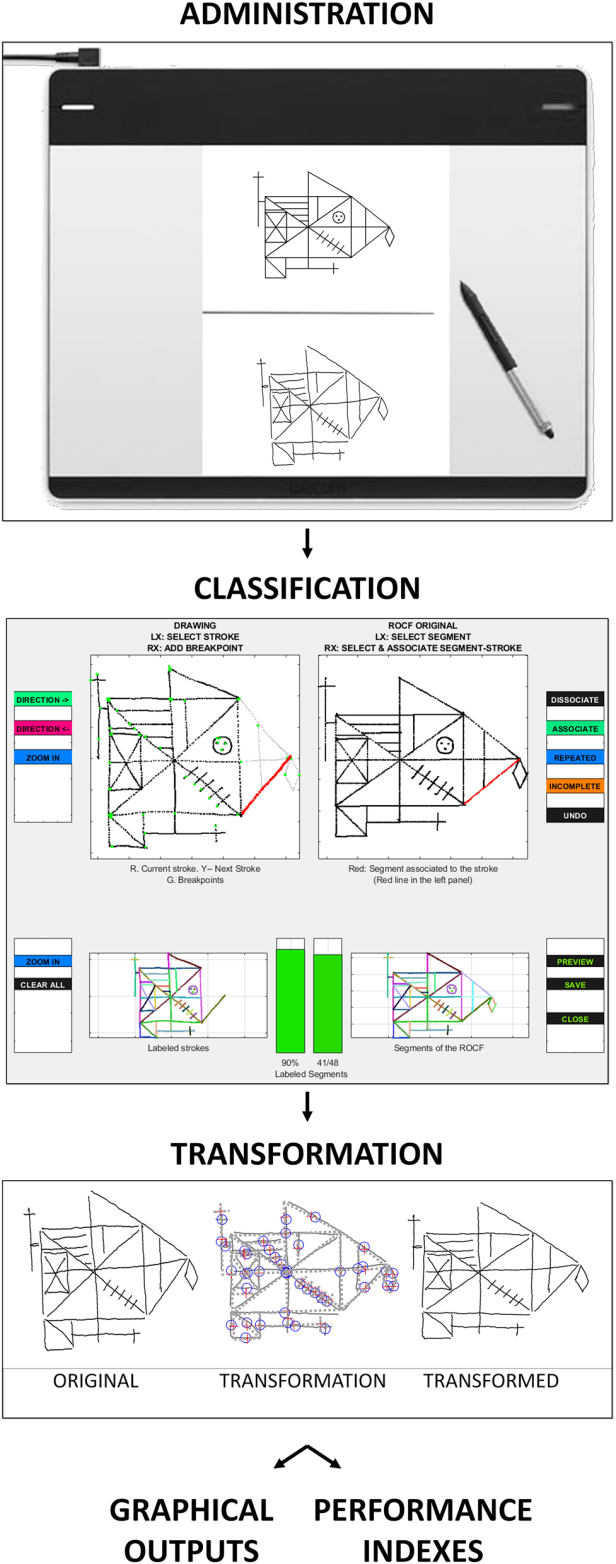
Steps of the T-RCF system. (1) Administration: during the copy of the T-RCF (i.e., Tablet-based Rey complex figure), every pen-surface contact is recorded over time via a graphics tablet connected to a computer. (2) Classification: after administering the task, a classification program is adopted to assist the manual classification of the segmented strokes into the 48 basic elements of the T-RCF. (3) Pre-processing: in this stage, the digital recording of the copy is subjected to a transformation process aimed at eliminating global distortion of the copy output. (4A) Graphical outputs: the T-RCF system produces three graphical outputs: a reproduction of the final graphic result (i.e., graphic output), a visual representation of the velocity profile (i.e., velocity output), and a visual representation of the drawing sequence (procedure output). (4B) Performance indexes: finally, the T-RCF scoring system computes the 12 indices of performance. The figures are created using Matlab (version 2017b, The Mathworks, Natick, MA, USA, www.mathworks.com) and Microsoft PowerPoint (Microsoft Office 365 Version 2105; www.office.com).

To address these limitations, we propose a novel Tablet-based digital system for the assessment of the Rey–Osterreith Complex Figure copy task (namely, T-RCF), which provides the opportunity to extract a variety of parameters from the entire process of drawing (for a similar tablet-based approach effectively applied for other purposes or to other drawing tasks see^24–29^). Specifically, the T-RCF automatically extracts a variety of indices of performance conceived, from a theoretical standpoint, to capture one of the following three dimensions underlying the execution of the RCF-copy task: (1) a spatial dimension expected to capture visuo-constructional skills, and precisely the success in forming the two-dimensional figure (i.e., RCF) by putting together its basic elements (for the T-RCF, basic elements consist of 44 single segments, 3 points and one circle which form the geometric units of the RCF; see Fig. 1A); (2) a procedural dimension mainly capturing information on the use of perceptual organisation strategies; (3) a kinematic dimension capturing aspects of movement control in handwriting and drawing^30,31^. Principal Components Analysis (PCA) was then applied to provide an empirical assessment of the indices' dimensionality and to assist in reducing the whole set of indices into a smaller set of composite scores^32–35^. Specifically, the identified structure was used to compute composite spatial, procedural, and kinematic scores. Their validity as measures of constructional, organisational, and motor ability was assessed by analysing their relationship with conventional scores of the RCF-copy task (both measures of copy accuracy^5^ and copy strategy^36^) and with several measures from a battery of other paper-and-pencil drawing tests selected to pose a variable load on constructional, planning, and motor skills.

## Methods

### Participants

For PCA purposes, the T-RCF test was administered to 102 healthy adults (55 females and 47 males; 49–84 years old, mean 63.0, SD 9.0; 98 right-handed; 5–23 years of education, mean 11.8, SD 4.0). The sample size for the PCA was selected in order to reach approximately 100 observations^34^. In order to provide initial evidence for the validity of T-RCF scores, a subsample of 35 participants (i.e., the first 35 participants who were enrolled; 15 males and 20 females; 49–76 years old, mean 65.1 years, SD 8.7; 34 right-handed; 8–19 years of education, mean 12.5, SD 3.4) was also tested with an extended battery of drawing tasks. In this case, the sample size was chosen to ensure sufficient power to detect meaningful correlations with medium/large size effect^37^ (> 0.45 for two-tailed correlations and > 0.40 for one-tailed correlations) at 80% power and 5% significant level. All participants were recruited as part of a larger ongoing project in collaboration between Fondazione Don Carlo Gnocchi-ONLUS IRCCS S. Maria Nascente and the University of Milano-Bicocca starting from 2013. The inclusion criterion for all participants was a normal score on a test of global functioning (i.e., MMSE^38^—adjusted scores > 23.8 according to the Italian normative data by Measso et al.^39^—or MoCA^40^—adjusted scores > 15.5 according to the Italian normative data by Santangelo et al.^41^). Moreover, all participants reported that they had no current or previous neurological or psychiatric disorders. Finally, all participants had normal or corrected-to-normal vision. This research was conducted in accordance with the guidelines outlined in the Declaration of Helsinki and was approved by the local ethical committee of Fondazione Don Carlo Gnocchi. Informed consent was obtained from each participant.

### Materials

The battery included the following drawings tasks selected to pose a variable load on constructional, planning, and motor skills:

- RCF-copy^4,5^: apart from digital scores, RCF conventional scores of constructional and organisational abilities were also obtained: (1) RCF-copy accuracy: as a conventional measure of visuo-constructional ability, the Rey–Osterrieth 36-point system was used^5,10–12,42^. This evaluates, in two-point scales, the accuracy, distortion, and location of the reproduction of each of the 18 geometric units (see Fig. 1) (total score: 0–36). (2) RCF-copy strategy: as a conventional measure of organisational performance, the Savage Scoring System^36,43^ was used, which evaluates five organisational units of the RCF-copy, each of which is required to be drawn as an unfragmented unit in order to receive points for organisation (total score: 0–6).
- RCF-recall^4,5^: this task requests the recall of the RCF ten minutes after the administration of the copy^42,44^. Although it is primarily a visual memory test, it also involves the same constructional and organisational functions as the RCF-copy. Accordingly, we computed the same conventional measures as in the RCF-copy (i.e., RCF-recall accuracy^5,10–12^ and RCF-recall strategy^36^).
- Clock drawing test (CDT)^2,45,46^: the CDT is a screening tool involving visual-constructional, numerical sequencing, and planning abilities. According to the Shulman variant^47^, the examinee is presented with a pre-drawn circle and is asked to draw a clock and the hands to indicate “ten minutes past 11 o’clock”. Placement of the numbers around the circle requires visual-spatial, numerical sequencing, and planning abilities. From this test, two measures were extracted: (1) CDT accuracy: an accuracy score was computed following the Shulman methodology^47^, which place emphasis on spatial accuracy (total score ranges: 1 = Perfect clock to 6 = Inability to make any reasonable representation of the clock). (2) CDT sequence: a qualitative measure of strategical sequencing in which the drawing is classified as spatially organised if the examinee adopts a quadrant-based strategical sequencing (i.e., highly organised strategy in which the clock numbers 12, 3, 6 and 9 are placed first subdividing the circle into four quadrants, and then the other numbers are added on in relation to these quadrats)^48^. All other approaches are classified as non-quadrant.
- Copy tasks battery: the battery included 21 heterogenous copying tasks selected from various neuropsychological assessment to cover a broad range of level of difficulty and constructional requirements in copying tasks^49–53^. Specifically, it included:
  - Nine stimuli (i.e., geometrical figures) from the test of Constructional Apraxia of Spinnler and Tognoni^54^ and the study of Arrigoni and De Renzi^50^;
  - Seven stimuli from the Bender–Gestalt Test^51^;
  - Two stimuli (i.e., one unreal and one real silhouette) from the Visual Object and Space Perception Battery (VOSP)^52^;
  - Two stimuli (i.e., one inanimate and one animate object) from the Snodgrass and Vanderwart dataset^53^;
  - One three-dimensional complex stimulus from a non-standardised database.
    Copy battery accuracy was scored by following a conventional procedure indicated in the Test for Constructional Apraxia^49^. This scoring methodology is particularly suitable to our needs as it is originally designed to extract a constructional score from a battery with various drawings of heterogeneous difficulty. Accordingly, for each drawing, 2 points were assigned in the case of perfect reproduction, 1 point was assigned in the case of partially incorrect reproduction (i.e., incorrect but still recognisable), and 0 points were assigned in the case of incorrect or unrecognisable reproduction. Finally, a single score was calculated from the sum of the scores assigned to each reproduction of the figures administered. Since the copying test consisted of 21 models, the score range was 0–42 points.
- Luria motor task: a copying task adapted from Luria’s figures^55,56^ was used whose characteristics and task requirement instruction place a significant load on motor skills. The model to copy consists of linear figures composed of different basic units and a line connecting them (see Figure S1). In this task, participants are instructed to use a predetermined procedure, and spatial accuracy is not considered. In order to place a significant load on motor skills, participants are instructed to copy the model with no interruption (i.e., not to raise the pen from the paper while copying), and the task is administered in three different motor variants. In the first condition, the model is printed at the top of the page and participants are required to copy the stimuli proceeding horizontally from left to right starting from a black dot located in the bottom-left section of the sheet. In the second condition, the model is again printed at the top of the page, but the copy is required to be made proceeding in the unusual direction of right to left, starting from a black dot located in the bottom right section of the sheet. In the third condition, the model is printed at the bottom of the page, and the copy is required to be made proceeding from left to right, starting from a black dot located in the top left section of the sheet. The total duration in the three conditions is computed as a measure of motor skills.

To summarise, four conventional measures of copy accuracy (i.e., RCF-Copy Accuracy, Copy Battery Accuracy, CDT Accuracy, and RCF-Recall Accuracy) were calculated from the drawing battery. Although these measures differ in terms of cognitive functions, they all have the common aspect of being heavily influenced by visuo-constructive skills. In addition, three conventional measures of the procedural organisation were calculated (i.e., RCF-Copy Strategy, RCF-Recall Strategy, and CDT Sequence). Finally, a measure of motor control was considered (i.e., Luria Motor Task).

### Procedure

All participants were individually tested in a quiet room. The T-RCF-copy was first administered to all participants. The procedure used for the administration of the T-RCF-copy is the same as the conventional one. In addition, in this case, the drawing process was recorded over time, employing a laptop computer connected via a USB to a graphics tablet (Wacom Intuos 2, Germany). Each test, printed on an A4 sheet of paper, was placed on the digitiser tablet in front of the examinee. The figure was printed in the upper half of the sheet of paper. The participant was required to copy the figure in the lower half part of the sheet. An ad-hoc ink pen (Wacom Ink Pen, Germany) for the copy was provided to the participant. For the entire duration of the task (i.e., from the first to the last pen and paper contact), the drawing process was recorded over time in terms of position and time of the tip of the pen at any pen-surface contact. After the administration of the T-RCF-copy, a sub-sample of participants was then tested with the extended battery of drawing tasks. The RCF-recall was administered first. Thus, the sheet of paper with the copied RCF was replaced with a blank one and, after 10 min (following the procedure used in the studies of Bertolani et al.^44^ and Caffarra et al.^42^), the participant was instructed to reproduce the RCF from memory. The delay interval was filled with an auditory sustained attention task (Elevator Counting Task, ECT^57^). After the RCF-recall, the CDT^58^, the extended battery of copying task and the Luria Motor Task were administered. Each copying task was administered following the same procedure adopted for the T-RCF-copy. Two qualified psychologists carried out conventional scoring for each drawing task. Average scores for each participant in each drawing task were then utilised in the analysis. In order to detect even small differences in our neurologically healthy group, for the measures of accuracy we purposefully adopted a fairly strict approach by penalising even small deviations from the expected drawing result. Note, that this approach leads to comparatively underestimate participants scores which are consequently hardly comparable to the normative values from the reference population. A synthetic description, including the means and standard deviations for each measure included in the drawing battery is reported in Table 1.

**Table 1:** Descriptive statistics (mean, and standard deviation) for each measure included in the drawing battery.

*INDEX*	*Mean*	*SD*	
RCF-Copy accuracy	30.77	3.02	Accuracy score for the direct copy of the RCF^5,43^ (> score > accuracy)
RCF-Copy strategy	3.77	1.72	Organisational score for the direct copy of the RCF^36^ (> score > organisation)
RCF-Recall accuracy	15.36	5.55	Accuracy score for the delayed recall of the RCF^5^ (> score > accuracy)
RCF-recall strategy	3.63	1.63	Organisational score for the delayed recall of the RCF^36^ (> score > organisation)
Copy Battery accuracy	29.31	3.50	Accuracy score for a battery of 21 copying tasks^50–54^ (> score > accuracy)
Luria motor task	180.69	45.59	Total duration for completing Luria Motor Task drawings^56,57^ (> value < motor control)
CDT accuracy	1.71	0.84	Accuracy score for the CDT^48^ (< score > accuracy)
CDT sequence	Quadrant N = 16	Non-quadrant N = 19	Qualitative measure of strategical sequencing in the CDT^49^ (quadrant = organized strategy)

For RCF-copy scores, N = 97; for other scores, N = 35.

*SD* standard deviation, *RCF* Rey Complex Figure, *CDT* Clock Drawing Test.

### T-RCF system

The ad-hoc software and any other code implementing algorithms described in the present paper are implemented in Matlab 2017b (The Mathworks, Natick, MA, USA; www.mathworks.com), and employs functions from the following toolboxes: *Optimization Toolbox* (Version 8.0)^59^, *Signal Processing Toolbox* (Version 7.5)^60^, *Statistics and Machine Learning Toolbox* (Version 11.2)^61^, and *Image Processing Toolbox* (Version 10.1)^62^. All the analyses implemented in the T-RCF system are based on the following information as input: time progression for each pen-down event (i.e., pen in contact with the drawing surface); the time-by-time position of the pen along the horizontal and vertical axes for each pen-down event; time-by-time indexing of initial pen-down events. Figure 2 summarises the main steps of the proposed T-RCF system. The T-RCF software and a manual detailing the instructions for the T-RCF is openly available on the Open Science Framework (https://osf.io/rt4hp/).

#### T-RCF stroke classification

The first step of the T-RCF analysis consists of an ad-hoc strokes classification procedure (see Fig. 2). This procedure aims to assist and facilitate the manual classification of the segmented strokes into 48 basic elements, as classified in Fig. 1A. The program first automatically segments the whole drawing into essential strokes (i.e., pen traces delimited by a pen-down and the following pen-up). When needed, manual segmentation of strokes into multiple sub-strokes is also allowed by the program. This is helpful in cases where a single stroke implements multiple elements that need to be classified as distinct parts of the T-RCF. Then the classification procedure allows selecting and associating each segmented drawing portion to a basic element of the T-RCF by clicking with the mouse, first, on the stroke to be classified and then on the corresponding T-RCF element. Specifically, for each segmented stroke, the classification procedure allows one of the following options to be selected: (a) to classify the stroke as belonging to one of the 48 basic elements of the original T-RCF; (b) to identify the stroke as belonging to an incomplete or repeated element; (c) to keep the stroke as unclassified in case of unrecognisable element (i.e., elements not unequivocally classifiable in one of the 48 basic elements of the T-RCF model).

#### T-RCF pre-processing

For the computation of spatial indices, only correctly reproduced (i.e., recognisable, complete, and not repeated elements) and linear (i.e., 1–24,29–48 basic elements Fig. 1A) elements are considered. As a preliminary step for the computation of the spatial indices, the digital recording of the copy is subjected to a transformation process aimed at eliminating global distortion of the copy output (overall size, inclination and placement of the copy) while maintaining the spatial relationships between its constitutive parts as unchanged. During this process, the measured drawing is globally rotated, horizontally and vertically rescaled and repositioned to minimise the difference between the actual and expected position of the T-RCF elements as measured from an ad-hoc template of reference. Therefore, such a transformation allows measures of spatial relationship between the constitutive elements of the T-RCF-copy to be extracted, regardless of the presence of global distortion of the figure as a whole (i.e., global errors of inclination, positioning, or sizing). An illustration of this process is shown in Fig. 2.

For the computation of the procedural indices, unrecognisable elements were excluded, and the order of reproduction of the remaining parts of the T-RCF was considered.

For the computation of kinematic indices, the entire drawing was considered. The velocity profile was digitally smoothed with a fourth-order Butterworth filter with a cut-off of 7 Hz^22^. The velocity profile in each stroke was then considered. Near zero-crossings in the velocity profiles (< 5 mm/s) were used to define the limits of the beginning and the end of each stroke. Only strokes exceeding a length of 10 mm were included in this analysis.

#### T-RCF graphical output

Once the strokes of the drawing are classified into their corresponding basic elements and the transformation process has been performed, the T-RCF scoring system produces three graphical outputs aimed at providing qualitative indications of the various aspects of the copy execution. More specifically, they consist of (1) a reproduction of the final graphic result (i.e., graphic output); (2) a visual representation of the velocity profile (i.e., velocity output); (3) a visual representation of the drawing sequence (procedure output). Examples of graphical outputs are shown in Fig. 6.

#### T-RCF performance indexes

Finally, the T-RCF computes 12 indices of performance conceived to capture a spatial, a procedural, and a kinematic dimension underlying the execution of the T-RCF-copy task (in order to facilitate their identification, spatial indices were prefixed with a lowercase “s", procedural indices with a lowercase “p" and kinematic indices with a lowercase “k").

The spatial dimension considers those spatial parameters that are crucial in preserving the shape of the figure as a whole, namely the relative placement, length and inclination of the basic elements of the figure reproduced by the examinee. Accordingly, the spatial indices are computed by comparing the transformed drawing and the corresponding reference figure.

- *Horizontal placement accuracy (sHP)* and *vertical placement accuracy (sVP)*: these two indices evaluate the degree of accuracy in preserving the relative horizontal (i.e., sHP) and vertical (i.e., sVP) placement of the basic elements of the T-RCF. sHP and sVP are calculated as the standard deviation of the placement of each element from its model placement. The position of each element is determined by taking the midpoint of each element (i.e., the point that is located on the exact midpoint of the two endpoints). Higher values correspond to lower accuracy in reproducing the spatial arrangement of the elements.
- Length accuracy (sLG): this index evaluates the degree of accuracy in preserving the relative size of the basic elements of the T-RCF. sLG is calculated as the standard deviation of the length of the elements from their correct length. Higher values correspond to lower precision in reproducing the correct proportions of the elements of the figure.
- Inclination accuracy (sIC): this index evaluates the degree of accuracy in preserving the relative inclination of the basic elements of the T-RCF. sIC is calculated as the standard deviation of the inclination of the elements from their correct inclination. Higher values correspond to lower precision in reproducing the relative inclination of the elements of the figure.

The T-RCF scoring system extracts four procedural indices aiming at quantifying on a continuous scale the degree of use of an organised constructional strategy. According to previous studies, the perceptual organisation is reflected in the drawing sequence and the degree of fragmentation of the elements of the T-RCF^63–68^. Here, the degree of use of an organised strategy was conceptualised along a continuum. At one extreme, a highly organised procedure consists of organising the figure into meaningful perceptual units and copying them according to their relative relevance. Individuals who use a perceptually organised copying strategy rely more on the hierarchical structure of the figure, beginning the task from the most important organisational unit, the Base Rectangle (green lines in Fig. 1C), which, together with the Main Substructure (blue lines in Fig. 1C), represents the guiding structures of the copy^63,69^. The Inner Details (i.e., red lines in Fig. 1C) are conversely the last part of the drawing and, together with the Outer Configurations (yellow lines in Fig. 1C), correspond to the secondary elements of the figures^63,68^ (see Fig. 1C). At the other extreme, a drawing strategy is absent. In this case, the drawing sequence is disorganised, the geometric units are reproduced without any consideration of their respective relevance, and the elements of the figure tend to be fragmented into multiple parts^3,36,43,63–65,68,70,71^. Following this premise, the T-RCF scoring system extracts four continuous procedural indices aimed at quantifying the degree of use of an organised copying strategy:

- Base rectangle priority (pBR): this index measures the level of priority given to the most relevant unit of the figure, namely, the base rectangle. It calculates the average time point (pen-up time points are excluded from this analysis) in which the examinee was employed in reproducing the base rectangle. Time points along the drawing progression are expressed on a scale from 0 (i.e., time point corresponding to the first pen-paper contact) to 100 (i.e., time point corresponding to the last pen-paper contact). The higher the value of *pBR,* the lower the priority given to the Base Rectangle.
- Inner details priority (pID): this index measures the level of priority given to the less relevant unit of the figure, namely, the inner details. Like pBR, pID calculates the average time point (pen-up time points are excluded for this analysis) in which the examinee was involved in drawing an element of the inner details of the figure. The higher the value of *pID,* the lower the priority given to the Inner Details.
- Organisation by relevance (pOR): this index measures the degree of the use of an organised order based on the relevance of the elements of the RCF. It is calculated as the number of times that participant interrupts drawing elements of primary relevance (i.e., Base Rectangle and Main Substructure) in order to copy elements of secondary relevance (Outer Configurations and Inner Details). The higher the value of pOR, and the lower the organisation in the order of reproduction of primary and secondary elements.
- Fragmentation (pFR): this index measures the degree of fragmentation of the basic elements of the RCF into multiple pieces. It is calculated as the number of times the reproduction of the units of the RCF is interrupted in order to reproduce elements belonging to other units. The higher the value of *pFR,* the higher the level of fragmentation of the units of the figure into multiple pieces.

Finally, the kinematic dimension includes four indices derived from the continuous tracing of the pen tip. These indices capture aspects of movement control in handwriting and drawing, which are altered in patients with movement disorders, such as those exhibited in Parkinson’s disease^30,31^. From the profiles of velocity obtained, the T-RCF scoring system extracts four kinematic indices of performance:

- Mean velocity (kVL): this index is focused on the rate of position changes over time which is one of the main kinematic features of the drawing. It is computed by averaging the mean velocity per stroke. The higher the value of kVL, the higher the movement control.
- Mean acceleration (kAC): this index measures the rate of velocity changes over time during the acceleration phases and is thus related to the forces exerted to propel the pen (i.e., Acceleration, kAC). The value of mean acceleration (i.e., rate of change of velocity with time > 0) per stroke is first determined, after which kAC is determined by averaging their absolute values across the strokes. The higher the values of kAC, the higher the movement control.
- Mean deceleration (kDC): this index (i.e., Deceleration, kDC) measures the rate of change of velocity during deceleration phases (i.e., stopping forces exerted during acceleration). The value of mean deceleration (i.e., rate of change of velocity with time < 0) per stroke is first determined, after which kDC is determined by averaging their absolute values across the strokes. The higher the values of kDC, the higher the movement control.
- Number of peak velocity (kPK): this kinematic index is an indicator of the fluency of the movement. It measures the average number of peaks in the velocity profile of the strokes, namely the number of times that a decrease follows an increase in the velocity profile. Ideally, the velocity profile of a fluent stroke is characterised by a unique inversion of the velocity profile (i.e., velocity would grow to a maximum peak and then decay until the end of the stroke). Less fluent movements are reflected by a larger number of peaks^30^.

A synthetic description, including the means and standard deviations for each index, is reported in Table 2.

**Table 2:** Descriptive statistics (mean and standard deviation) and synthetic description of T-RCF indices.

INDEX	Mean	SD	Synthetic description
*sHP*	2.77	1.48	Accuracy in positioning the elements on the horizontal axes (> sHP < accuracy)
*sVP*	2.61	1.12	Accuracy in positioning the elements on the vertical axes (> sVP < accuracy)
*sLG*	4.49	1.61	Accuracy in reproducing the length of the elements (> sLG < accuracy)
*sIC*	5.67	2.06	Accuracy in reproducing the inclination of the elements (> sIC < accuracy)
*pOR*	8.93	4.65	Level of organisation in the order of the elements of the T-RCF (> pOR < organisation)
*pBR*	18.81	11.11	Level of priority given to the base rectangle (> pBR < priority)
*pID*	73.97	10.35	Level of priority given to the inner details (> pID < priority)
*pFR*	6.11	4.09	Degree of fragmentation of the basic units of the T-RCF (> pFR > fragmentation)
*kVL*	38.26	9.83	Mean velocity per stroke (> kVL > velocity)
*kAC*	243.09	99.18	Change of rate of velocity during acceleration phases (> kAC > change)
*kDC*	252.39	106.24	Change of rate of velocity during deceleration phases (> kDC > change)
*kPK*	4.57	1.56	Number of peaks per stroke (> kPK > number of peaks)

N = 97(excluding outliers); *sHP* horizontal placement accuracy, *sVP* vertical placement accuracy, *sLG* length accuracy, *sIC* inclination accuracy, *pBR* base rectangle priority, *pID* inner details priority, *pOR* organisation by relevance, *pFR* fragmentation, *kVL* mean velocity, *kAC* mean acceleration, *kDC* mean deceleration, *kPK* number of peak velocity.

### Statistical analyses

For each participant, the 12 index scores were extracted using the T-RCF scoring system. Participants with index scores of greater than four standard deviations from the variable means (N = 5) were excluded from the analysis. This conservative threshold for outlier detection was chosen following the rule of thumb suggested by Hair et al.(^34^, p. 90) for sample sizes larger than 80. In order to reduce the whole set of indices into a smaller set of composite scores, indices were standardised, and PCA with orthogonal varimax rotation of the loading matrix was applied^32–35,72^. Kaiser–Meyer–Olkin Measure of Sampling Adequacy^34,73,74^ (MSA) and Bartlett's test of sphericity^72,75^ were performed to examine the appropriateness of the datasets for PCA. Results from Horn's parallel analysis^72,76^ (i.e., Monte Carlo approach comparing observed eigenvalues with those identified in a distribution of eigenvalues from PCAs of 1000 random data sets with the same size generated randomly) and Kaiser’s criterion^77^ (i.e., approach retaining components with eigenvalues greater than 1) were considered to evaluate the appropriateness of our theoretical assumptions regarding the existence of three distinct dimensions in our index scores. Communalities were assessed for each index, with communalities below 0.5 taken to indicate items poorly accounted for by the factor solution^34^. Primary loadings were considered meaningful if they exceeded 0.50^34^. Indices with loadings exceeding ± 0.30 on two or more components were considered as cross-loading indexes^34^. In order to simplify the model structure as much as possible cross-loading indexes were not retained. Composite scores were extracted for each component using a cumulative scale constructed by taking the average of the indices loading on that component^32,34^. Negative loadings were reverse-scored before creating the composite scores. The reliability of the cumulative scales was measured by Cronbach's alpha^78^ with values of 0.60 to 0.70, deemed the lower range of acceptability^34,79^. Split-sample analysis was applied to assess the robustness of the solution across the sample. Thus, the entire sample was randomly split into two equal parts of 49 participants each, and the PCA was re-estimated for both in order to test for comparability (for details on the split-sample procedure, see Hair et al.^34^ p. 176).

The relationships among composite scores and between composite scores and demographic variables were assessed using Pearson-correlation coefficients^72^. Construct validity of the composite scores was evaluated via partial correlation analysis (using Pearson-correlation coefficient)^72^ between composite scores and scores from the extended battery of drawing tasks controlling for age and education. One-tailed correlations were performed in those cases in which we had a precise prediction about the direction of the effect (i.e., positive correlations between spatial composite score and constructional drawing measures; positive correlations between procedural composite score and organisational drawing measures; a negative correlation between kinematic composite scores and motor measure of drawing). Two-tailed correlations were performed for all other cases. Finally, to assess the independent contribution of each composite score to RCF-copy accuracy, a linear multiple regression analysis was performed, controlling for age and education.

All statistical analyses were conducted in RStudio (Version 1.4.1106)^80^. The following packages were used for statistical analyses and visualisation: *psych* (version 2.1.3)^72^; *dplyr* (version 1.0.5)^81^; *ltm* (version 1.1)^78^; *stats* (version 4.0.5)^82^; *corrplot* (version 0.88)^83^; *qgraph* (version 1.6.9)^84^.

## Results

### Component analysis results

**Figure 3:**
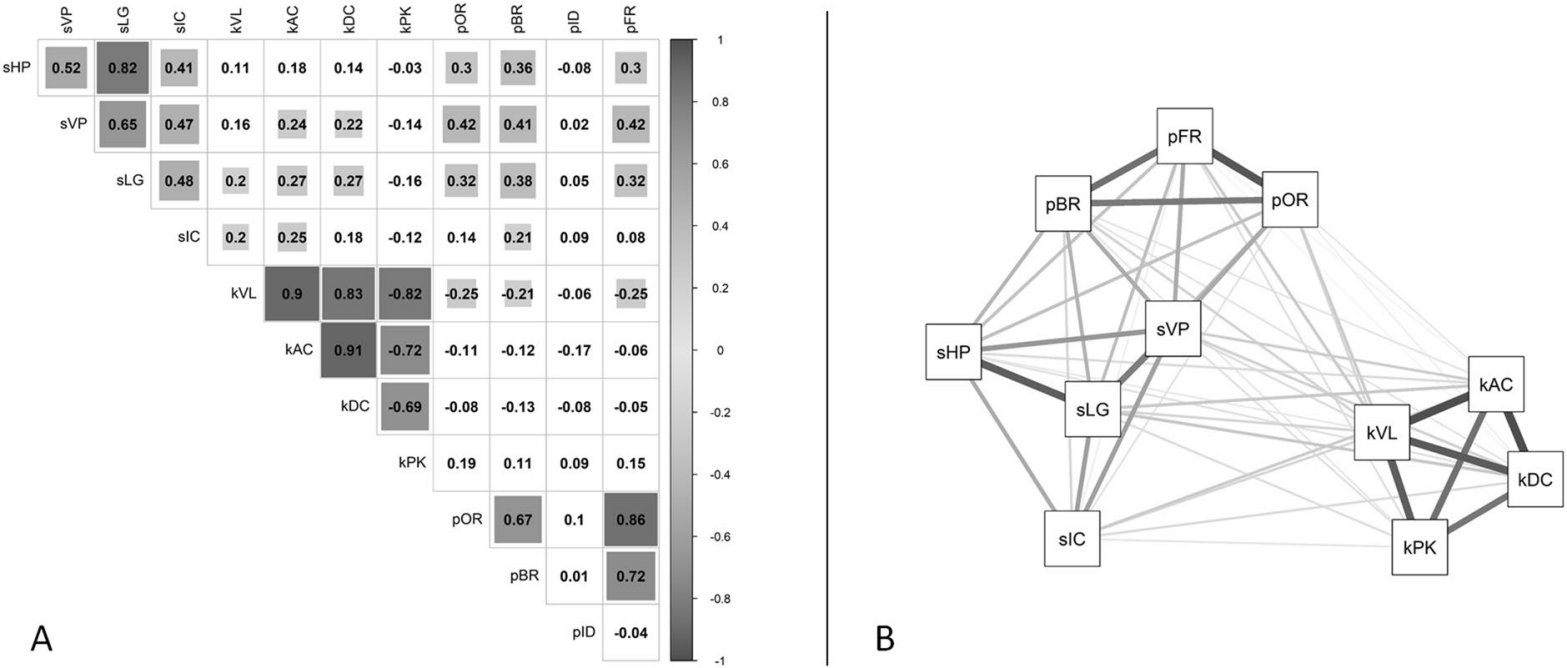
Correlations among the T-RCF index variables. (**A**) Correlation matrix within the T-RCF (i.e., Tablet-based Rey complex figure) index variables. Significant (p < 0.05) correlations are highlighted in greyscale according to the level of correlation (from white for r = 0 to dark grey for the highest level of correlation). The figure is created in RStudio (Version 1.4.1106)^80^ using the package corrplot (version 0.88)^83^. (**B**) Visual representation of the pattern of correlations among the T-RCF index variables (excluding pID). The nodes represent the variable indices. Nodes are arranged such that more highly correlated variables are closer to one another. Correlation levels are indicated by the tone (from white for r = 0 to dark grey for r =  ± 1) and by the width of the lines connecting the nodes (i.e., the larger the width of the lines, the higher the level of correlation). The figure is created in RStudio (Version 1.4.1106)^80^ using the package qgraph (version 1.6.9)^84^. *sHP* horizontal placement accuracy, *sVP* vertical placement accuracy, *sLG* length accuracy, *sIC* inclination accuracy, *kVL* mean velocity, *kAC* mean acceleration, *kDC* mean deceleration, *kPK* number of peak velocity, *pBR* base rectangle priority, *pID* inner details priority, *pOR* organisation by relevance, *pFR* fragmentation.

For the initial solution of the PCA, the minimum amount of data was satisfied, with a sample size of 97, providing a ratio of 8.1 observations per variable^34^. Initially, the appropriateness of PCA was examined. The correlation matrix (Fig. 3A) indicated high redundancy in the data. All the variables correlated at least 0.3 with at least one other item except for pID, which was not correlated with any other variable. Likewise, an inspection of the MSA for pID revealed a value well below the accepted level of 0.5^34,73,74^ (i.e., pID MSA = 0.19) and, therefore, was discarded from subsequent analyses (for a visual representation of the resulting pattern of correlations, see Fig. 3B). After removing pID, all the MSA for both the overall test and each index were above the acceptable level of 0.50 (i.e., overall MSA = 0.76; minimum MSA for individual indices = 0.64)^73,74^. In addition, the result of Bartlett's test of sphericity^75^ was highly significant (χ^2^ (55) = 886.15, p < 0.001), confirming the presence of significant correlations among several indices. These results suggest that PCA is an appropriate procedure to reduce the original indices into a smaller number of composite scores^33–35^.

**Figure 4:**
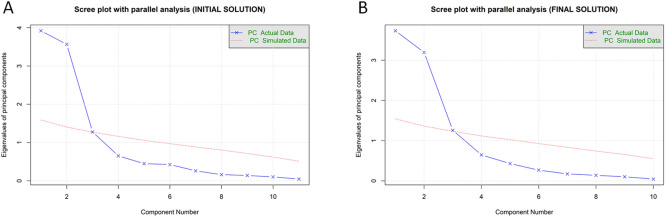
Scree plots with parallel analyses. (**A**) Scree plot (the line with × s) showing eigenvalues of the initial principal component analysis (PCA) and parallel analysis with 100 simulations (dashed line). (**B**) Scree plot (the line with × s) showing eigenvalues of the final PCA and parallel analysis with 100 simulations (dashed line). The figures are created in RStudio (Version 1.4.1106)^80^ using the function fa.parallel of the package psych (version 2.1.3)^72^. *PC* principal component.

PCA with orthogonal varimax rotation of the loading matrix was then performed on the remaining 11 index variables. Horn's parallel analysis for PCA^76^ left uncertainty about the existence of three components (observed eigenvalues: C1 = 3.93, C2 = 3.57, C3 = 1.27; simulated eigenvalues: C1 = 1.58, C2 = 1.41, C3 = 1.27; Fig. 4A): observed eigenvalues for the first two components were well above those for the simulated randomly generated datasets (ΔC1 = 2.19, ΔC2 = 1.85), whilst the eigenvalues for the third component was roughly equivalent to that of the randomly generated datasets (ΔC3 = − 0.0002). However, Kaiser’s criterion^77^ clearly supported the existence of three components in line with our theoretical assumptions. Thus, three components were retained. Together, the three components accounted for 80% of the variance in total scores. Specifically, the first three components explained 35.7%, 32.5%, and 11.6% of the variance, respectively.

**Figure 5:**
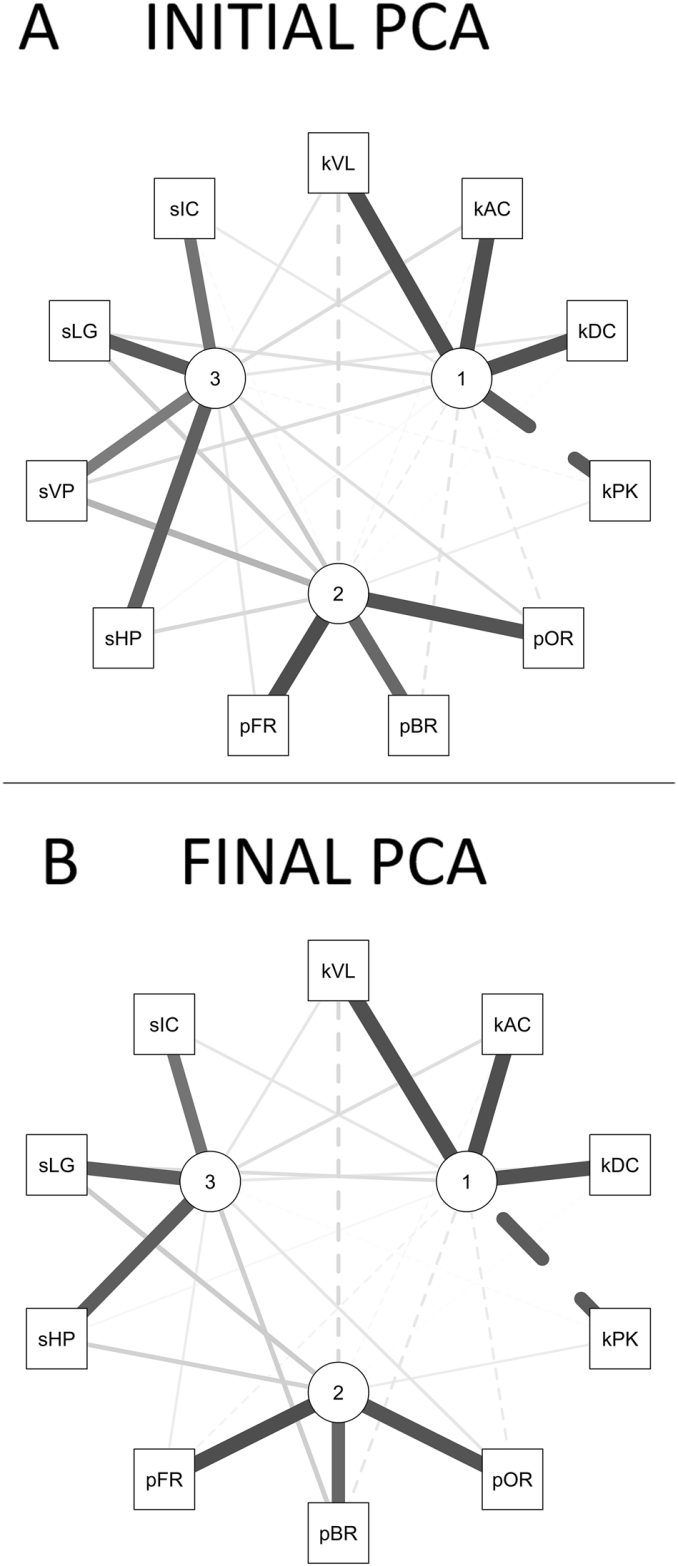
Relationship between indices and components. Visualisation of the relationship of the index variables and the three components extracted from the initial principal component analysis (PCA) (**A**) and the final PCA (**B**) solutions. The index variables are indicted by three-character abbreviations and the components by numbered nodes. Nodes are arranged in a way that they are closer to their more highly correlated index variables. Loading levels are indicated by the tone (from white for loading = 0 to dark grey for the highest level of loading) and the width of the lines connecting the index variables to the three components. The figures are created in RStudio (Version 1.4.1106)^80^ using the package qgraph (version 1.6.9)^84^. *sHP* horizontal placement accuracy, *sVP* vertical placement accuracy, *sLG* length accuracy, *sIC* inclination accuracy, *pBR* base rectangle priority, *pOR* organisation by relevance, *pFR* fragmentation, *kVL* mean velocity, *kAC* mean acceleration, *kDC* mean deceleration, *kPK* number of peak velocity.

The results for the PCA (Table 3A; Fig. 5A) showed no index whose component loading was less than 0.4. However, this solution showed sVP to cross-load on more than one component. In particular, the analysis suggests that sVP loads not only with the other spatial indices (i.e., sHP, sLG and, sIC) on component 3 but also on what appears to be a procedural component (i.e., component 2), based on common loading with the procedural indices (i.e., pOR pBR, and pFR). Thus, for the final stage, sVP was removed and then the PCA was recalculated^34^. A PCA of the remaining ten index variables was conducted using varimax orthogonal rotation. Both Kaiser’s criterion^77^ and Parallel Analysis^76^ confirmed the retention of the first 3 components (observed eigenvalues: C1 = 3.74, C2 = 3.21, C3 = 1.25; simulated eigenvalues: C1 = 1.54, C2 = 1.36, C3 = 1.22; Fig. 4B), explaining 82% of the variance (C1 = 37%, C2 = 32%; C3 = 12% of variance). The results are reported in Table 3B and Fig. 5B. An examination of the component matrix shows that all the indices exhibit a communality higher than 0.5. No index met the criterion for a cross-loading with two loadings greater than ± 0.30. Significant primary loadings (i.e., >  ± 0.50) emerged for all the indices included in the final PCA, and all of them also exceeded ± 0.70, thus indicating a well-defined structure of the PCA^34^.

**Table 3:** Standardised loadings (pattern matrix) based upon correlation matrix for the three components (C1, C2, and C3) solution using orthogonal (i.e., Varimax) rotation of the loading matrix for the initial principal component analysis (PCA) (panel A) and the final PCA (panel B).

	C1	C2	C3	h2	u2	com
**(A)**	**INITIAL PCA**					
sHP	0.03	0.20	**0.84**	0.75	0.25	1.1
sVP	0.17	0.39	**0.69**	0.66	0.34	1.7
sLG	0.16	0.24	**0.87**	0.83	0.17	1.2
sIC	0.11	− 0.03	**0.74**	0.56	0.44	1.0
kVL	**0.94**	− 0.20	0.13	0.93	0.07	1.1
kAC	**0.94**	− 0.04	0.17	0.92	0.08	1.1
kDC	**0.92**	− 0.01	0.13	0.87	0.13	1.0
kPK	− **0.87**	0.08	− 0.03	0.76	0.24	1.0
pOR	− 0.11	**0.91**	0.16	0.86	0.14	1.1
pBR	− 0.13	**0.80**	0.26	0.73	0.27	1.3
pFR	− 0.07	**0.94**	0.12	0.91	0.09	1.0
**(B)**	**FINAL PCA**					
sHP	0.04	0.24	**0.86**	0.80	0.20	1.2
sLG	0.17	0.27	**0.86**	0.84	0.16	1.3
sIC	0.12	0.00	**0.74**	0.56	0.44	1.1
kVL	**0.94**	− 0.20	0.12	0.93	0.07	1.1
kAC	**0.94**	− 0.04	0.16	0.92	0.08	1.1
kDC	**0.93**	− 0.01	0.12	0.87	0.13	1.0
kPK	− **0.87**	0.09	− 0.02	0.76	0.24	1.0
pOR	− 0.10	**0.92**	0.13	0.87	0.13	1.1
pBR	− 0.12	**0.81**	0.25	0.74	0.26	1.2
pFR	− 0.06	**0.95**	0.09	0.91	0.09	1.0

**(C)**	**SPLIT-SAMPLE 1**			**SPLIT-SAMPLE 2**		
	**C1**	**C2**	**C3**	**C1**	**C2**	**C3**
sHP	0.11	0.20	**0.91**	0.11	0.20	**0.91**
sLG	0.21	0.22	**0.88**	0.21	0.22	**0.88**
sIC	0.11	0.04	**0.76**	0.11	0.04	**0.76**
kVL	**0.94**	− 0.19	0.18	**0.94**	− 0.19	0.18
kAC	**0.94**	− 0.01	0.17	**0.94**	− 0.01	0.17
kDC	**0.96**	− 0.02	0.03	**0.96**	− 0.02	0.03
kPK	**− 0.85**	0.01	− 0.14	**− 0.85**	0.01	− 0.14
pOR	− 0.06	**0.92**	0.10	− 0.06	**0.92**	0.10
pBR	− 0.08	**0.78**	0.21	− 0.08	**0.78**	0.21
pFR	− 0.03	**0.95**	0.11	− 0.03	**0.95**	0.11

Primary loadings >  ± 0.40 are shown in bold. Secondary loadings >  ± 0.30 are underlined. The column "h2" contains the component communalities (i.e., the amount of variance in each index variable explained by the components). The column "u2" contains the component uniquenesses (i.e., the amount of variance not accounted for by the components—or 1–h2). The column "com" reports the Hoffman's index of complexity for each item (i.e., the number of latent components required to account for the observed variables)^88,89^. Panel C) Validation of component analysis by split sample estimation with Varimax rotation. The two tables report standardised loadings (pattern matrix) based upon correlation matrix for the three components (C1, C2, and C3) solution using Varimax oblique rotations of the loading matrix for the two samples. Primary loadings >  ± 0.40 are shown in bold. The column "h2" contains the component communalities (i.e., the amount of variance in each index variable explained by the components).

*sHP* horizontal placement accuracy, *sVP* vertical placement accuracy, *sLG* length accuracy, *sIC* inclination accuracy, *pBR* base rectangle priority, *pOR* organisation by relevance, *pFR* fragmentation, *kVL* mean velocity, *kAC* mean acceleration, *kDC* mean deceleration, *kPK* number of peak velocity.

Split-sample analysis was then applied^34^. Table 3C contains the final solution for the split-sample results. As can be seen, the solutions obtained for the two samples are comparable in terms of loadings, thus indicating that the model results are stable within the sample.

A meaningful name was attributed to each component describing its content. The first component showed high positive primary loadings for kVL, kAC, kDC, and kPK and was named “Kinematic” (KIN). In particular, kVL, kAC, and kDC loaded positively on KIN while kPK loaded negatively on KIN, indicating that lower values of KIN are indicative of greater movement control. The second component was named "Procedural" (PRO). In particular, pOR, pBR, and pFR loaded positively on PRO, indicating that lower values of PRO are indicative of greater use of organised strategy. Finally, the third component showed high positive primary loadings for sHP, sLG, and sIC indices and was termed "Spatial" (i.e., SPA). Specifically, lower values of SPA are associated with higher accuracy in reproducing the spatial relationship of the constituent elements of the T-RCF-copy.

Finally, composite scores were extracted for each component (i.e., SPA, PRO, and KIN). Given its negative loading, the kPK index was reverse-scored before creating the KIN composite score. Finally, the SPA and the PRO composite scores were reversed scored so that higher scores represented better performances for all the composite measures. The assumption of dimensionality for each cumulative scale was supported by the clean interpretation of each component in the model’s results (i.e., indices were strongly associated with each other and represented a single component)^34,85^. Cronbach's alpha was above the recommended level of 0.70 for all scales (i.e., 0.8 for the scale of SPA composite score; 0.95 for the scale of the KIN composite score; 0.9 for the scale of the PRO composite score)^34,79^. Correlation analyses for the composite scores showed that SPA score is positively correlated with PRO score (r = 0.35; p < 0.001) and negatively correlated with KIN score (r = − 0.22, p = 0.027). No significant correlation emerged between PRO and KIN (r = 0.17, p = 0.096).

### Relations between automated and conventional measures of drawing

Initially, we explored the relationships between T-RCF composite scores with age and education (Table 4). All three indices exhibit a small to medium significant correlation with education. Specifically, the higher the level of education, the better the visual constructional performance as measured by SPA score (r = 0.31, p = 0.002). Similarly, the better the procedural organisation as measured by PRO score (r = 0.25, p = 0.013) and the higher the education, the better the motor control as measured by KIN score (r = 0.23, p = 0.026). Moreover, SPA exhibits medium significant correlations with age (SPA: r = − 0.41, p < 0.001). Specifically, increasing age is associated with lower constructional performance as measured by SPA score. No correlation between age and PRO score (r = 0.02, p = 0.869) and between age and KIN score (r = 0.06, p = 0.589) emerged.

**Table 4:** Correlation coefficient (r) between T-RCF (i.e., Tablet-based Rey Complex Figure) composite scores (i.e., SPA, PRO, and KIN) with demographic data (i.e., age, education) and conventional scores extracted from the battery of drawing tasks (adjusted for age and education).

	Demographic		RCF-COPY		Other constructional			Other organisational		Motor
	Age	Educ	RCF-Copy accuracy	RCF-Copy strategy	Copy-Batt accuracy	CDT accuracy	RCF-recall accuracy	CDT sequence^A^	RCF-recall strategy	Luria motor task
SPA	− 0.41***	0.31**	0.73***	0.19	0.53**	− 0.50**	0.41**	0.26	0.10	− 0.13
PRO	0.02	0.25*	0.47***	0.85***	0.09	− 0.10	0.27	0.44**	0.71***	0.09
KIN	0.06	0.23*	− 0.34**	0.12	− 0.35*	− 0.01	0.06	0.17	0.18	− 0.43**

Highest correlation for each conventional score is shown in bold. Correlations with age, education, and RCF-copy scores, N = 97; correlations with other scores, N = 35; * p < 0.05; ** p < 0.01; *** p < 0.001; ^**A**^point-biserial correlation with 0 for non-quadrant strategy and 1 for quadrant strategy.

*RCF* Rey complex figure, *CDT* clock drawing test, *CDT* clock drawing test, *SPA* spatial composite score, *PRO* procedural composite score, *KIN* kinematic composite score.

Subsequently, in order to provide clear insights regarding the construct captured by each T-RCF composite score (i.e., T-RCF SPA, PRO, and KIN), we analysed their correlations with drawing scores extracted via conventional scoring methodologies (controlling for age and education) (Table 4). SPA composite score consistently correlates with conventional measures of spatial accuracy (i.e., RCF-Copy Accuracy, Copy Battery Accuracy, CDT Accuracy, RCF-Recall Accuracy). Specifically, the highest correlation emerges between SPA score and the conventional visual-constructional measure of RCF-copy accuracy (r = 0.73; p < 0.001). Correlations between SPA and the other constructional measures are also moderate (SPA and copy battery accuracy: r = − 0.53, p < 0.001; CDT accuracy and SPA: r = 0.50, p = 0.001; RCF-recall accuracy and SPA: r = − 0.41, p = 0.007). Therefore, the better the SPA score, the better the performance in other constructional measures. The correlations between SPA and the other conventional measures of drawing were all low and not significant (RCF-copy strategy: r = 0.19, p = 0.057, CDT sequence: r = 0.26, p = 0.131; RCF-recall strategy: r = 0. 10, p = 0.556; Luria motor task: − 0.13, p = 0.462).

Similarly, PRO composite score consistently correlates with conventional measures of procedural organisation. The highest correlation emerged between PRO score and RCF-copy strategy (r = 0.85; p < 0.001) followed by the correlation between PRO and RCF-recall strategy (r = 0. 71; p < 0.001). Point-biserial correlation between PRO and CDT-Sequence was also significant (r = 0.44; p = 0.004), indicating that a greater use of organised strategy in the T-RCF is associated with the adoption of a quadrant-based strategical sequencing in the CDT. Besides procedural measures, PRO exhibits a significant correlation with RCF-copy accuracy (r = 0.47; p < 0.001) suggesting that the conventional measure of RCF-copy accuracy is also influenced by procedural aspects of the task. PRO does not exhibit any other significant correlation with conventional measures of drawing (copy battery accuracy: r = 0.09, p = 0.589; CDT accuracy: r = − 0.10, p = 0.581; RCF-recall accuracy: r = 0.27; p = 0.117; Luria motor task: r = 0.09, p = 0.592).

Finally, KIN composite score exhibits a moderate positive correlation with the motor performance measured in Luria Task (r = 0.43, p = 0.005). Moreover, KIN also negatively correlates with performance for RCF-Copy Accuracy (r = − 0.34, p < 0.001) and Copy Battery Accuracy (r = − 0.35, p = 0.04), indicating that kinematic aspects of drawing also affect drawing accuracy. The other correlations between KIN and conventional measures of drawing are low and not significant (CDT accuracy: r = − 0.01 , p = 0.976; RCF-recall accuracy: r = 0.06, p = 0.742; RCF-copy strategy: r = 0.12, p = 0.253; CDT-sequence: r = 0.17, p = 0.324; RCF-recall strategy: r = 0.18, p = 0.308).

Finally, to assess the contribution of each dimension of the T-RCF to RCF-copy accuracy, we ran a regression analysis including, as dependent variable, RCF-Copy Accuracy and, as predictors, SPA composite score, PRO composite score, and KIN composite score (including education and age as covariates). The results showed that all three composite scores significantly predicted RCF-copy accuracy. Specifically, SPA was the strongest predictor of RCF-copy accuracy (t (91) = 7.689, p < 0.001), followed by PRO (t (91) = 4.218, p < 0.001) and, KIN (t (91) =  − 2.991, p = 0.003) scores.

### Qualitative analysis of the graphical output

**Figure 6:**
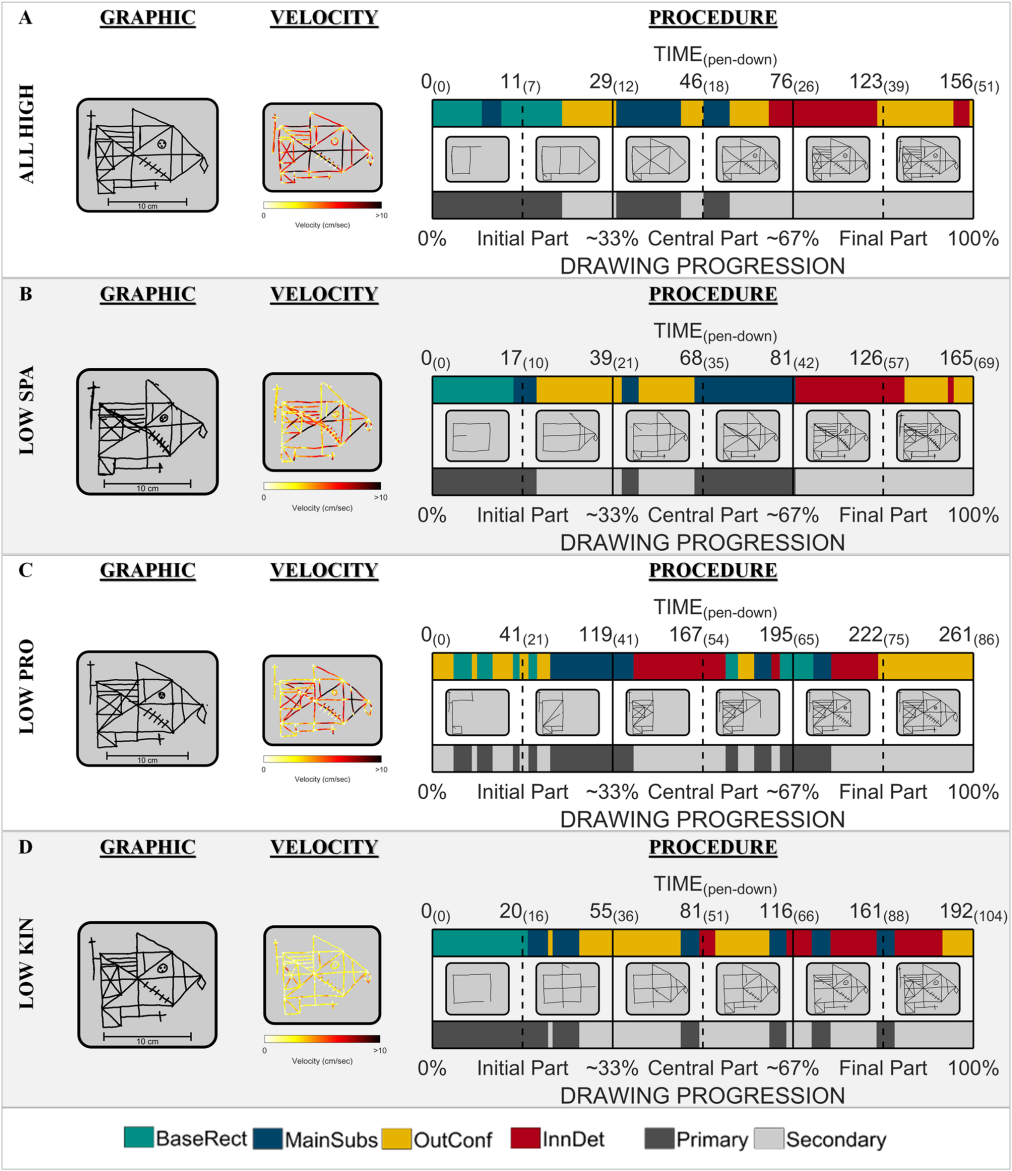
Graphical output of the T-RCF. The figure shows examples of graphical outputs for the T-RCF (i.e., Tablet-based Rey complex figure). (**A**) The graphical output for a participant achieving a high measure for all the composite scores. (**B**) The graphical output for a participant achieving a low SPA score. (**C**) The graphical output for a participant achieving a low PRO score. (**D**) The graphical output for a participant achieving a low KIN score. On the left of each one, graphical outputs of the final results are reported. In the middle, the graphical outputs of the velocity are reported. Each point of the strokes is coloured from light yellow (~ 0 velocities) to dark red (> 10 cm/s). On the right, the procedure outputs are reported. From top to bottom, the procedure outputs report: (a) the elapsed time (in seconds) at six different time frames equidistant along with the progression of the drawing (i.e., from the left to the right, when ~ 17%, ~ 33%, 50%, ~ 67%. ~ 83% and 100% of the total drawing length was reproduced). The time progression for the drawing phases only (i.e., pen-down time) is shown in brackets. (b) A coloured bar indicating the drawing progression according to the structure that the participant draws at each instant (i.e., base rectangle = GREEN; main substructures = BLUE; outer configurations = YELLOW; inner details = RED). (c) the drawing timeline panel showing the progress in the task at six time frames of the drawing (i.e., from the left to the right, when ~ 17%, ~ 33%, 50%, ~ 67%. ~ 83% and 100% of the total drawing length were reproduced). (d) A black and grey bar showing the drawing progression according to the relevance of the element that the participant draws in each drawing point (i.e., primary = BLACK; secondary = GRAY). (e) the drawing progression shown as a percentage from 0% (i.e., first pen-paper contact) to 100% (i.e., last pen-paper contact). The figure is created in Matlab (version 2017b, The Mathworks, Natick, MA, USA, www.mathworks.com).

Figure 6 provides qualitative views of the constructs captured by the three components. The first figure on the top (Fig. 6A) reports the graphical output for a participant achieving high measures for all the composite scores (all > 0.5 SD from the group mean). Each image reports the graphical outputs from participants selectively achieving a low SPA (A), low PRO (B) or low KIN (C) score (< − 0.5 SD from the group mean).

As can be seen in panel B of Fig. 6, low SPA is accompanied by a degradation of the constructional result. In this specific case, the observation of the final result is sufficient to reveal the presence of degradation of the spatial relationships between the constituent elements of the T-RCF: the elements in the right half of the figure are shifted to the right, with consequent deformation of the subunits in the right half (i.e., upper triangle and right triangle) and elongation of the elements on the left of the figure. It is to be noted that an abnormal SPA score can be quantified on the drawing final result, independently from the progression of the drawing itself.

However, the only observation of the final result cannot reveal the presence of procedural and kinematic alterations in the performance of the other two participants (Fig. 6, panel C,D). Panel C demonstrates the presence of disorganisation in the procedural sequence adopted by the participant. By looking at the coloured and the black-and-grey bars of the procedural sequence from left to right, it is evident that there are continual colour changes and colour repetitions. This indicates that the constitutive parts of T-RCF have been fragmented during copying and that the various units have been reproduced without low consideration of their respective relevance. Accordingly, the basic rectangle was completed in the final part of the task; most of the inner details were drawn during the central part of the task; the sequence order is based on the proximity of the lines rather than the relevance of the elements.

On the other side, Panel D highlights the presence of an alteration in kinematic aspects. In this case, the velocity profile tends to remain consistently slow (light yellow) with only a few episodes of increased speed (shades tending to red/black). This indicates slowed movements with no noticeable velocity changes throughout the task that may suggest the presence of a lower level of motor control.

## Discussion

In order to consider various aspects potentially affecting the final result of the RCF-copy, in this study, we have implemented a novel Tablet-based assessment (i.e., T-RCF), acquiring data and information for the entire execution and extracting several indices that capture various dimensions of the drawing process. The T-RCF was administered to a group of healthy adults along with a paper-and-pencil drawing battery, from which constructional, procedural, and motor measures were obtained. Initially, a PCA was used to convert the whole set of indices of the T-RCF into a smaller set of meaningful composite scores. This analysis provided useful insight into the structure of relationships between the various dimensions of drawing considered. Specifically, it confirmed the existence of distinct components in the execution of the T-RCF-copy. The PCA identified three distinct dimensions in the whole set of T-RCF indices of performance. One dimension covers such variables which reflect spatial accuracy in the copy (i.e., SPA) and, specifically, accuracy in the length, inclination, and placement of the elements on the horizontal axes. A second dimension covers procedural aspects with respect to the use of perceptual organisation strategies and specifically regarding the order of drawing and the level of fragmentation in the elements of the T-RCF-copy (i.e., PRO)^63–68^. A third dimension covers kinematic aspects of the velocity profile (i.e., KIN), which are known to characterise movement control in handwriting and drawing^30,31^.

The PCA results provided support for the computation of a composite score for each dimension (SPA, PRO, and KIN score). Correlation analyses involving these scores and demographic data indicated an age-related decline in SPA score. The influence of education was observed for all three composite scores with higher performance with increasing education. In addition, correlation analyses provided clear insight into the constructs captured by the composite scores. SPA score is associated with conventional RCF-copy score, as well as with spatial accuracy in other drawing tasks. Although the tasks that were adopted here to measure spatial accuracy are very heterogeneous in their format and cognitive requirement, they all have the common property of being influenced by constructional skills. Therefore, these results provide SPA with converging evidence of validity as a measure of visual constructional ability. At the same time, our analysis showed correlations between PRO and other measures of procedural organisation in drawing, thus providing evidence for its validity. Besides conventional measures of strategy in the RCF-copy and recall, this measure was also associated with the type of strategical sequencing of the clock numbers in the DCT task. This suggests the generalizability of such an organisational aspect with respect to different types of procedural requirements (i.e., number sequencing). Finally, KIN was found to be related to the measure of motor control used in this study. Overall, correlational analyses provided insights into the constructs captured by the three composite scores. The SPA, PRO and KIN composite scores appear to be valid estimates of constructional, organisational, and motor performance in this task.

At this point, it is important to note that all three composite scores were correlated and predicted RCF-copy accuracy score. This indicates that all three of these dimensions somehow affect the overall spatial accuracy as measured in the final graphic product. This study demonstrates the importance of considering the distinct contribution of each drawing dimension to draw valid and specific conclusions which cannot be derived from the paper-and-pencil score of copy accuracy. Problems both at a constructive and/or organisational level can compromise the final result. In addition, motor aspects have been found to influence the final graphic product. However, in this case, it is important to note that a negative association between KIN and spatial accuracy in RCF-copy emerged (but also between KIN and SPA score). Initially, this finding appears challenging to explain. In other studies, the kinematic indices from which KIN score is calculated were impaired in the presence of movement control disorders such as in Parkinson’s disease^22,30,31^. Accordingly, a positive effect of high motor performance on the spatial accuracy of the copy would be expected, and not vice versa. However, it is essential to consider that the group selected in this study consisted of healthy participants (without motor control difficulties). In this context, it is plausible that unusually high KIN performance conveys a different meaning. In fact, high KIN scores can be found in the performances of those participants who can execute the task with high velocity, producing significant accelerations and decelerations and producing a low number of peaks in velocity. This pattern of velocity is not uniquely an indication of high movement control, which by itself is expected to influence spatial accuracy positively. However, it may also suggest the use of a hasty and careless drawing style which should accordingly impair spatial precision. On the other hand, low KIN scores in healthy adults may suggest a more meticulous and careful performance style. In other words, this pattern should indicate a sort of speed-accuracy trade-off in copying figures. Like in movement^86,87^, there are trade-offs in drawing, in which an individual may globally sacrifice velocity for accuracy, or vice-versa and the two terms are consequently related in a way that as the speed of movements increases, their spatial accuracy decreases. Having a complete picture of the execution of the drawing, and therefore of both kinematic and spatial aspects, is essential to draw valid conclusions and separate inaccurate spatial performance due to visuospatial problems or carelessness in drawing.

Interestingly, the preliminary PCA analysis also provided valuable intuitions into the relationships between various aspects of drawing. These analyses indicated that sVP, namely, the level of accuracy in positioning the elements on the vertical axes, loaded not only on the spatial component but also on the procedural component. This result revealed that the level of accuracy in positioning the elements on the vertical axes is not a specific aspect of spatial accuracy as it may also indicate alteration at a procedural level. This indicates that different processes underly the identification of the correct position of the elements along the vertical and horizontal drawing plane. It is worth noting that placing an element on the vertical axes is a different and more demanding cognitive task than performing the same operation along the horizontal axes. In fact, in this task, the copy is typically reproduced under the reference model. In this condition, the horizontal coordinates of the reference model and the drawing plane are the same. This aspect allows a vertical line intersecting a point on the reference model to be “simply” imagined in order to find its corresponding position on the horizontal axes of the drawing plane. Conversely, there is no correspondence between the vertical coordinates of the drawing and the model plane. For the vertical axis, it is necessary to rely on other spatial information to infer the correct position of an element of the figure, such as the available drawing area, the position of the other elements which have already been drawn, and their reciprocal spatial relationships. It is, therefore, plausible that the adoption of an effective procedural strategy simplifies this cognitive operation. The adoption of an organised procedure wherein configural elements (e.g., base rectangle) are drawn first, provides the participant with supporting frames of reference for each element which is subsequently drawn (which is added on in relation to them). This would explain why the accuracy in placing elements on the vertical axis is not a specific spatial index as it may be the result of an interplay between spatial and procedural skills.

Another interesting and unexpected result emerged for pID, the procedural index measuring the priority given to inner details. Literature indicates that when an organised constructional strategy is used, details are the last part of the drawing^63,68^. However, in this study, correlations among the T-RCF index indicated that the priority given to inner details of the RCF was not associated with the other procedural variables. However, we can hypothesize that this type of index may still represent an effective indicator of procedural alterations plausibly in the presence of more severe organizational deficits. Conversely, in our study with healthy individuals, this type of alteration, if present, might be too subtle to emerge, and this may explain why it appeared unrelated to other procedural indices. Further studies in clinical populations are needed to confirm this observation.

Finally, some limitations need to be considered. First, although the sample size of 35 participants who performed the entire battery allowed us to reveal the most meaningful relationships between composite scores and conventional drawing measures, a larger cohort would be required to ensure adequate power to detect and consider also smaller effects. Second, the scoring system is not fully automatic since it requires a preliminary manual stage of stroke classification into the elements of the reference figure. Although we implemented an easy-to-use program to assist the examiner in the classification, this requires time (about 5 min per drawing) and a minimum of training. A recent study by Webb et al.^28^, implemented a novel automated scoring algorithm for a digital complex figure copy task. This methodology was able to identify the drawing parts of the figure successfully. Future research can aim to implement a similar algorithm for the T-RCF.

In summary, this study confirms that the graphic product of drawing results from an interplay of multiple components. It has provided useful insight into the structure of relationships between various dimensions of drawing execution. The T-RCF offers a unique opportunity to extract performances’ scores from the full drawing about three main distinct dimensions involved in drawing, namely, spatial, procedural, and motor. Positive evidence for the validity of T-RCF composite scores is provided in this study. Furthermore, this novel screening technique also provides qualitative graphical output that may be useful for clinicians in adequately interpreting individual performance.

Further studies are required to establish the validity and reliability of the automated T-RCF scoring system before its adoption for clinical and diagnostic purposes. Furthermore, at the moment, the effectiveness of the T-RCF in isolating constructional, motor and organisational ability from the RCF-copy cannot be generalised with respect to the clinical population. However, the results of this study are auspicious in this sense. In fact, cognitive and motor variability in the healthy population is expected to be smaller than in the clinical. It is even more remarkable that, notwithstanding, the automated T-RCF-copy scoring system is sufficiently sensitive to capture variability in such behavioural data. This makes our findings very promising for the adoption of this tool in clinical populations and for diagnostic purpose.

## Supplementary Information

Supplementary Information 1.

## Data Availability

The T-RCF software and a manual detailing the instructions for the T-RCF is openly available on the Open Science Framework (https://osf.io/rt4hp/). The dataset generated and analysed during the current study is available from the corresponding author on request.
