# Supplementary material for: Automated scoring for a Tablet-based Rey Figure copy task differentiates constructional, organisational, and motor abilities
Source: Sci Rep. 2021 Jul 21;11:14895. doi: 10.1038/s41598-021-94247-9 (PMC8295394; doi:10.1038/s41598-021-94247-9)
Supplement: Supplementary file 1 — Supplementary Information 1. [file 41598_2021_94247_MOESM1_ESM.pdf]

Automated scoring for a Tablet-based Rey Figure Copy task differentiates constructional, organisational, and motor abilities

Marco A. Petilli[1*], Roberta Daini[1,2], Francesca Lea Saibene[3], Marco Rabuffetti[3]

1 Psychology, Università degli Studi di Milano Bicocca, Milan, Italy

2 NeuroMI - Milan Center for Neuroscience, Milan, Italy

3 IRCCS Fondazione Don Carlo Gnocchi ONLUS, Milan, Italy

*Corresponding author:

marco.petilli@unimib.it

University of Milano-Bicocca

Department of Psychology

Piazza dell'Ateneo Nuovo, 1 – 20126 Milan, Italy

Tel. +39 02 6448 3767

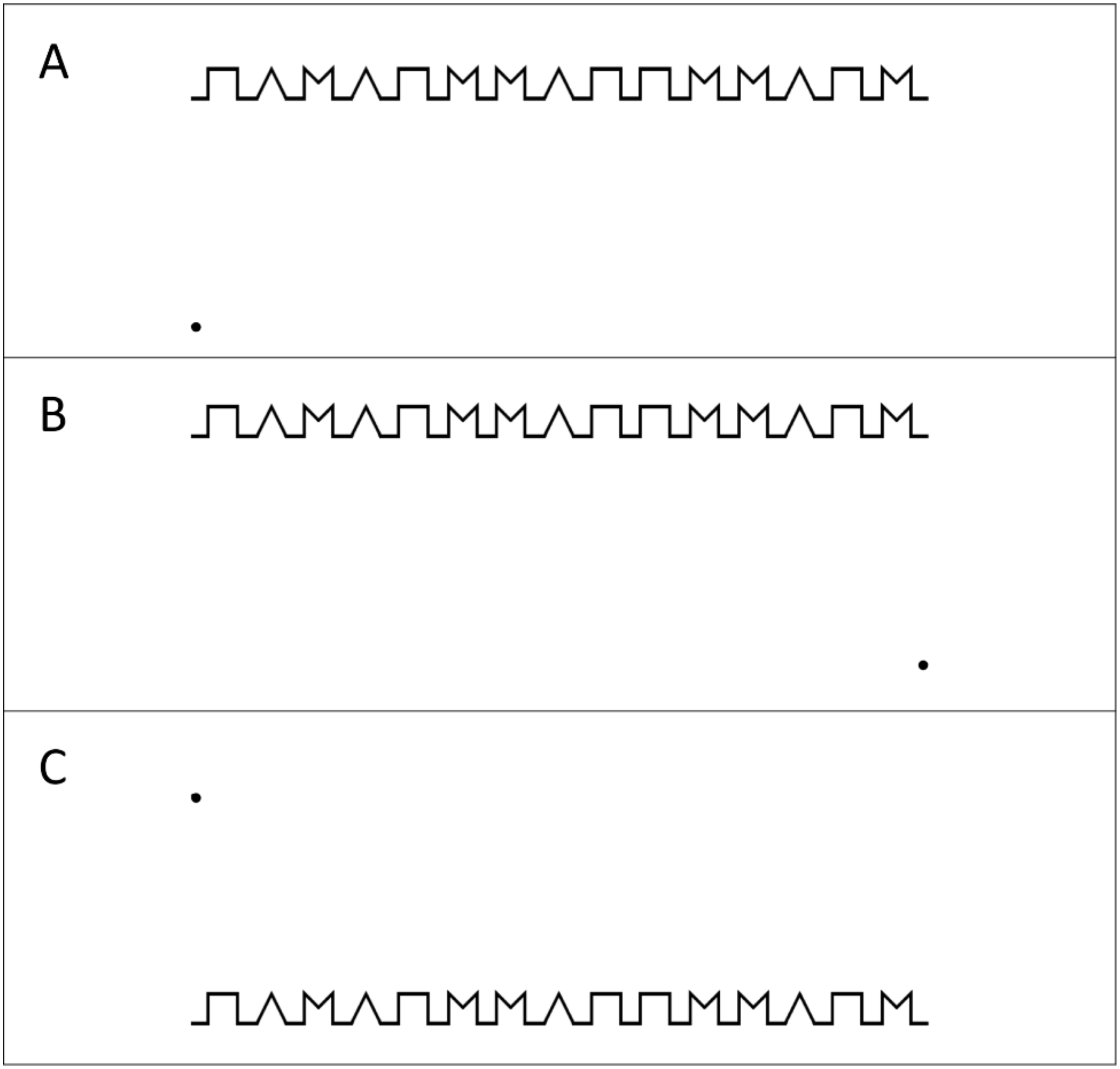

Supplementary Figure S1: Luria Motor Task. Figure created with Adobe Photoshop 7.0 (Adobe Corp; www.adobe.com/).
